# Human Polymorphisms as Clinical Predictors in Leprosy

**DOI:** 10.1155/2011/923943

**Published:** 2011-12-18

**Authors:** Ernesto Prado Montes de Oca

**Affiliations:** Pharmaceutical and Medical Biotechnology Unit, Research Center in Technology and Design Assistance of Jalisco State, National Council of Science and Technology, 44270 Guadalajara, JAL, Mexico

## Abstract

Genetic and serum markers in human host can predict leprosy susceptibility *per se* as well as be useful in classification and/or prediction of clinical variants and immunological responses in leprosy. Adequate and timely assessment of potential risks associated with these 38 host leprosy genes could diminish epidemiological burden and improve life quality of patients with this still prevalent mycobacterial disease.

## 1. Introduction

In recent years, evidence has accumulated for so-called human “multidisease susceptibility genes” that play a role in several infectious and noninfectious diseases [1], and genes presumably more specific on its clinical impact when dysfunctional, that probably do not predispose to the disease *per se* but to a clinical phenotype when the disease is already present [2]. A decline in leprosy research, probably for the erroneous belief that the battle is won, has left unanswered questions regarding the exact mechanism of disease infection and progression. Addressing these questions could help in the final eradication of leprosy [3].

Human leprosy (or Hansen disease) is a chronic granulomatous infectious disease caused by the obligate intracellular organism *Mycobacterium leprae* [4, 5]. Leprosy is still an important health problem worldwide, with the highest incidences in Asia, Africa, and Latin America [6]. In 2008, *∼*250,000 new cases of leprosy were reported to World Health Organization [7]. The high concordance rates for leprosy infection when monozygotic and dizygotic twins are compared (84.55% versus 18.35%, resp., media of two independent reports) [7] as well as loci that are linked or associated with leprosy, reveal a strong genetic component in the susceptibility and response to the infection with *M. leprae. *Furthermore, complex segregation analyses suggest an oligogenic model of leprosy susceptibility, with a few major genes influencing disease and several additional genes and variations causing subtle effects on disease outcome [8]. The origin of transmission of *M. leprae* is mainly from untreated lepromatous patients [9] and the people most probable infected (leprosy *per se*) are patient's contacts whose carry susceptibility alleles in loci 20p12 [10], *TNFA *[11], as well as *PARK2 *and* PACRG *genes [12]. Genetic variants in human host, mainly single nucleotide polymorphisms (SNPs) can predict susceptibility to leprosy *per se *and to clinical variants when leprosy is already present [2, 8, 13]. Even when death due to leprosy, caused by laryngeal obstruction, is uncommon [14], adequate and opportune detection of potential risks could diminish epidemiological burden and improve life quality of patients with this still unerradicated mycobacterial disease.

## 2. Leprosy Classification

### 2.1. Ridley-Jopling Classification

Once the infection in the patient is confirmed, it is important to classify leprosy in order to determine the appropriate treatment and predict risk of complications [9] as physical deformities, sensorial loss, and permanent nerve damage [14]. Since 1940, the drug to treat leprosy was dapsone, but today a multidrug scheme is recommended [14]. Ridley-Jopling classification uses histopathological and clinical features and the bacteriological index as well. It classifies leprosy in tuberculoid (TT), borderline tuberculoid (BT), borderline (BB), borderline-lepromatous (BL), and lepromatous (LL) categories. The different categories usually correlate with host immune response. Indeterminate leprosy is an earlier nondefined state [9, 15]. TT patients present a limited number of hypopigmented, anesthetic skin lesions with microscopically undistinguishable bacteria. The correlated T_H_1-cell-mediated immune (CMI) response (IL-2, IL-6, IL-12, IFN-*γ*, and TNF) promotes the formation of delineated granulomas—central areas of infected macrophages, often fused into multinucleate giant cells, surrounded by T cells—which suggested function in infection control is still controversial [16, 17] and data on *M. marinum* suggesting a role in infection dissemination seems interesting to test in human leprosy [18]. Conversely, LL cases present numerous sensitive or anesthetic skin lesions with high bacillary loads. LL if untreated may slowly progress to bacteremia [14]. The correlated T_H_2-antibody response (IL-4, IL-5, IL-10) impedes granuloma formation, allowing for uncontrolled bacterial replication and continuous infiltration of the skin and nerves. Borderline forms, that is, borderline-tuberculoid (BT), borderline (BB), and borderline-lepromatous (BL), comprise the majority of cases. These individuals present intermediate clinical and histological phenotypes resulting from immunologically unstable responses [7]. The incubation period between infection and clinical disease can vary from some months to up to 30 years, with a mean period of 4 years for TT and 10 years for LL [19].

### 2.2. WHO Operational Classification: Multibacillary and Paucibacillary

Another leprosy classification proposed by the World Health Organization (WHO) recognizes the paucibacillary (PB) and multibacillary (MB) forms, in which the patient presents with 1–5 skin lesions and 6 or more, respectively [20]. Patients with PB disease present very low bacterial counts, exhibiting localized infection and lesions characterized by expression of type 1 (T_H_1) T helper lymphocyte-related cytokines (cell-mediated immunity) as IL-2, IL-6, IL-12, IFN-*γ*, and TNF. Patients presenting MB form are more susceptible to the pathogen and exhibit systemic infection and lesions expressing T_H_2 cytokines (humoral immunity) [2, 7, 21]. Multibacillary leprosy roughly includes BB, BL, and LL forms, and paucibacillary the TT and BT forms [7, 21]. For a complete table of polymorphisms associated with leprosy types according to WHO classification, see Table 1.

## 3. Immunological Reactions and Nerve Damage in Leprosy

### 3.1. Mitsuda Response

The standard measure of cell mediated immunity (CMI) against *M. leprae* is the Mitsuda response [4], a delayed granulomatous skin reaction elicited by the intradermal injection of heat-killed *Mycobacterium leprae* formulations—lepromin is one example—and measured 21 to 28 days after inoculation [21]. Although the Mitsuda test has been developed to assess immune response to live *M. leprae *in natural conditions of infection, other microbial agents influence this test, such as live bacille Calmette-Guerin (BCG) vaccine, and *M. tuberculosis*. Therefore, the Mitsuda test reflects the ability to develop an immune granuloma after mycobacterial infection but is not a marker for specific immune responses to the leprosy bacillus and is not related to clinical variant in leprosy patients [13].

### 3.2. Type I Reactions: Reversal Reaction

Reversal reactions (RRs, oka type I reactions) represent the acute immune episodes of T_H_1 inflammatory response to *M. leprae *antigens that occur in skin and/or nerves and are the leading cause of neurological impairment in leprosy patients. The skin lesions become acutely inflamed and oedematous and may ulcerate. Oedema of the hands, feet, and face can also be a feature of a reaction, but systemic symptoms are unusual. They occur most frequently in borderline categories (BL, BT, BB) with a frequency of 30% in these patients [9] or in MB patients, and age is a relevant additional risk factor for both RR occurrence and sequelae after RR treatment [32]. RR are frequently recurrent and this can lead to nerve damage. RR can occur at any time but are frequently seen after starting multidrug therapy (MDT) or during the puerperium [9].

### 3.3. Type II Reactions: Erythema Nodosum Leprosum

Erythema nodosum leprosum (ENL) has a prevalence in leprosy patients of 24%. ENL is a systemic immune reaction which can occur in LL (up to 50% of LL cases) and borderline patients (9% of BL patients) and is postulated to be caused by extravascular deposition of immune complexes (ICs) resulting in neutrophil infiltration and complement activation. (See *C3* and *C4B* genes below.) There are three subclinical types of ENL, named single acute ENL (8% of cases), multiple acute ENL (repeated discrete episodes), and chronic ENL (continuous, longer, and more severe episodes, 62.5% of cases) [33]. ENL affects many organs with an acute onset but it can evolve into a chronic phase and it can be recurrent. ENL produces fever and in the skin painful tender red papules or nodules which occur in crops often affecting the face and extensor surfaces of the limbs. The lesions may be superficial or deep causing a panniculitis. Bullous ENL has been described and lesions may ulcerate. Subcutaneous tissue involvement may lead to tethering and fixation to joints causing loss of function. ENL reactions may also produce uveitis, neuritis, arthritis, dactylitis, lymphadenitis, and orchitis. The prolonged inflammation of organs can lead to blindness and sterility. A greater infiltration of the skin and a higher bacterial index are two relevant risks for developing ENL [9]. Improved strategies for treatment and management of these reactions need to be developed [33].

### 3.4. Nerve Damage

Peripheral nerve damage is an important issue in leprosy clinical complication that diminish quality of life of patients [34]. Nerve involvement in leprosy affects sensory, motor, and autonomic function of peripheral nerves. Acute neuritis leads to nerve function impairment, which if not treated timely and adequately leads to permanent loss of nerve function causing peripheral sensory and motor neuropathy [9]. The mechanism underlying nerve injury in leprosy is poorly understood [35]. TT patients present granulomatous inflammation of peripheral nerves causes palpable enlargement, which may or may not be painful and causes sensory and motor loss in the distribution of the affected nerve. There is enlargement and infiltration of fascicles, which may show caseous necrosis [9]. In LL patients, nerve function impairment occurs earlier than in TT patients [35] the destruction of dermal nerves leads to a glove and stocking neuropathy, peripheral nerve damage occurs late. There is bacterial proliferation within the Schwann cells, which leads to foamy degeneration of the cells losing their regeneration ability [9]. This nerve damage causes deformities and disabilities are a social stigma and cause discrimination of patients and their families [34].

## 4. Genes and Its Variants Associated with Leprosy Types and Immunological Reactions

### 4.1. Complement Component 3 (C3, 19p13.3-p13.2)

Human complement component forms ester linkages with hydroxyl groups. Cleavage of C3 exposes a reactive short-lived thioester moiety in C3b, which covalently attaches to amine and carbohydrate groups on the target surface. This initial tagging is quickly amplified on foreign cells but is immediately regulated on human cells. Moreover, the reactivity of the thioester moiety to specific carbohydrates might lead to preferential opsonization of foreign particles and represent a basic pattern recognition mechanism [36, 37]. In an extrinsic protease pathway, plasmin, thrombin, elastase, and plasma kallikrein can also cleave and activate C3. Furthermore, target-bound MBL can activate C3 independently of mannan binding lectin serine protease 2 (MASP-2), C2, or C4 *in vitro. *The highly abundant C3 can act as a systemic surveillance protein that has few endogenous ligands but becomes transformed into one of the most versatile binding partners upon activation to C3b. The remaining C3a is a powerful chemoattractant that guides neutrophils, monocytes, and macrophages toward sites of complement activation and induces TLR activation in antigen presenting cells [37]. Phenolic glycolipid 1, a major component of *M. leprae *cell wall and activated complement component C3, are exposed on the cell membrane of *M. leprae*-infected human dendritic cells. C3 costimulate naïve T cells via CD46 thus induce the differentiation of IL-10-secreting regulatory T cells. In this way, *M. leprae* subverts host natural immunity to provoke an adaptive response that favors bacillary survival [38]. In populations from Ethiopia and Mali, it was found that mean C3 serum concentration was lower in leprosy patients (0.88 mg/mL in Ethiopic and 0.73 mg/mL in patients from Mali) when compared with nonleprosy subjects (1.1 mg/mL, both populations) being the lowest C3 levels in each population found in BL patients (0.62 mg/mL) and in TT patients (0.81 mg/mL) from Mali and Ethiopic populations, respectively. Otherwise C3 phenotypes designated according to electrophoretic mobility FF (fast-fast), FS (fast-slow), and SS (slow-slow) were not associated with leprosy. Correlation of both variables were not reported [22].

### 4.2. Complement Component 4 B (C4B, 6p21.3)

In the classical pathway or antibody-dependent pathway of complement, C1s component cleaves C4 into C4a and opsonin C4b, thereby exposing a previously hidden thioester and leading to covalent deposition of C4b on surfaces in the immediate vicinity of the activation sites (opsonization) [37]. Deficiency of the C4b isoprotein with higher binding capacity for thiol groups has been found in association with Immuno Complexes (IC) diseases involving bacterial or fungal antigens. The non expressed allele C4B*Q0 increases risk of ENL probably because C4b is involved in opsonization of pathogens and immune complex clearance [39] interacting with C3b and CR1 (CD35) to promote neutrophil-mediated phagocytosis [37].

### 4.3. Collagen Type III, Alpha 1 (*COL3A1*, 2q31)

Type III collagen is a fibrillar-forming collagen comprising 3 *α*-1 chains and it is expressed throughout embryogenesis. In adult, type III collagen is an essential component of the extracellular matrix in a variety of internal organs and skin. Mutations in *COL3A1* gene cause type III and type IV Ehlers-Danlos syndrome, a condition of remarkable skin and joint plasticity that often leads to major blood vessels rupture in early adult life [40]. Patients homozygous for *250-bp COL3A1* procollagen III alpha I in Indian population were associated with the MB form of the disease [23]. A probable linkage with *SCL11A1* located at the neighbor locus 2q35 has been suggested [24].

### 4.4. Human *β*-Defensin 1 (*DEFB1*, 8p23.1)

The human *β*-defensin 1 (hBD-1) has been associated with different allergic and infectious diseases as well as cancer with a relevant biological function in protecting mothers against lactational mastitis and breast-fed infants from diarrhea. In addition, hBD-1 is capable of chemoattract human immature dendritic cells (iDCs) and memory T cells *in vitro*, promotes caspase-mediated apoptosis of cancer cells, and its expression correlates with insulin and glucose levels [41]. In a Mexican population *DEFB1* gene was associated with LL, the SNP *668C* alters one out of five putative binding sites for nuclear factor kappa B1 (NF-*κ*B1 or p50/p105) [2, 41] and functionally correlates with lower constitutive expression but surprisingly also with higher IFN-*γ*-dependent inducibility of both hBD-1 and hBD-3 [42]. Thus, diminished NF-*κ*B-dependent constitutive expression caused by *668C*, could be a risk factor for developing leprosy into LL been suggested that this allele is more permissive to *M. leprae *dissemination in patients already infected [2]. This probably explains the extremely rare heterozygote advantage *DEFB1* promoter [41, 43, 44] and recurrent overrepresentation of heterozygotes in this locus [45, 46].

### 4.5. Ficolin 2 (*FCN2*, 9q34.3)

Ficolin 2 (also called L-ficolin or hucolin) is a soluble pattern-recognition molecule that binds to different pathogen-associated molecular patterns (PAMPs), such as lipoteichoic acid, carbohydrates and acetylated groups, initiating activation complement through lectin pathway leading to pathogen phagocytosis [37]. Functional haplotypes of polymorphisms that produce normal ficolin-2 level protect against clinical leprosy, and low serum levels of circulating protein are clearly associated with promoter polymorphisms *−986, −602, −4* [47].

### 4.6. Major Histocompatibility Complex Class I and II (*HLA*, 6p21.3)

The main function of major histocompatibility complex (MHC) is antigen presentation of processed antigens to T cells. Each classical HLA class I gene encodes a transmembrane *α*-chain that noncovalently associates with *β*-2 microglobulin (*B2M*, 15q21–q22.2). The heterodimeric class I molecule, expressed on all nucleated cells, presents cytosolic pathogen-associated peptide to CD8^+^ T cells. In addition, the classical HLA class I molecules (HLA-A, -B, and -C) bind peptides of intracellular origin and present them to CD8^+^ T cells, engaging killer cell immunoglobulin-like receptors (KIRs) expressed on natural killer cells been this as NK “licensing” that render the death of* M. leprae*-infected cells [48]. The heterodimeric class II molecule, expressed on B cells and antigen-presenting cells, presents extracellular or intravesicular pathogen-associated peptide to CD4^+^ T cells (T_H_1 and T_H_2) resulting in cytokine production [7]. Polymorphisms in human leukocyte antigens (HLA)*-*class I (A, B, C) and class II (DR, DQ) have been associated with leprosy types. *HLA-DR* polymorphisms modulate the cytokine profile of CD4^+^ T from TT and LL forms against *M. leprae*-derived heat shock proteins [49]. HLAs polymorphisms in as HLA A*0206, A*1102, B*4016, B*1801, B*5110, Cw*0407, and Cw*0703 have been associated with leprosy and HLA A*0101, Cw*04011, and Cw*0602 were associated with protection in Indian population. Haplotype A*1102-B*4006-Cw*1502 was found in association with LL patients. Haplotypes A*1102-B*4006-Cw*0407 and A*0203-B*4016-Cw*0703 were increased in both LL and TT patients [50]. HLA-class I antigens *A9, A10, A32, B5, B21, Bw4, Bw6, Cw1, Cw2, *and HLA-class II antigens *DR9, DR10, DRw52, DQ1, DQ3* were associated with leprosy. HLA-class I antigens *A3, B44, B49,* and HLA-class II antigen *DQ5* were found protective in a Turkish population [51]. *HLA-B46* allele and *HLAB46/MICA-A5* haplotype protects against MB leprosy in Chinese population [52]. In Chinese and Indian populations, *HLA-DRB1/HLA-DQA1* locus, particularly HLA-DRB1*1501 was associated with leprosy [6]. Nevertheless, the interpretation of linkage and association studies of the HLA complex requires caution because of long-range LD [7].

### 4.7. Interferon Gamma (*IFNG*, 12q15)

Interferon-gamma (IFN-*γ*), or type II interferon, is a cytokine critical for innate and adaptive immunity against viral and intracellular bacterial infections and for tumor control. Aberrant *IFNG* expression is associated with a number of autoinflammatory and autoimmune diseases. The importance of* IFNG* in the immune system stems in part from its ability to inhibit viral replication directly, but most importantly it derives from its immunostimulatory and immunomodulatory effects. IFN-*γ* is produced predominantly by natural killer (NK) and natural killer T (NKT) cells as part of the innate immune response, and by CD4 and CD8 cytotoxic T lymphocyte (CTL) effector T cells once antigen-specific immunity develops [53]. Low levels of IFN-*γ* in response to *M. leprae* antigen in household contacts of MB patients predict leprosy infection [54]. In a Brazilian population, there was a protective effect for +*874T* SNP carriers in *IFNG, *a gene also previously reported to be associated with tuberculosis [55].

### 4.8. Interleukin 4 (IL-4, 5q31.1)

Interleukin-4 (IL-4), a T_H_2 cytokine, regulates expression of a C-type lectin, CD209 antigen (also called Dendritic Cell-Specific Intercellular Adhesion Molecule 3-Grabbing Nonintegrin, DC SIGN), and subsequent *M. leprae* internalization by Schwann cells [56]. IL-4 is expressed in skin lesions of MB patients [56] and its *−590 C* allele decreases leprosy susceptibility [57]. IL-4 has been shown to act downregulating TLR2 and IL-10 on monocytes. IL-10 suppresses production of IL-12 [9] and also downregulates antimycobacterial peptide cathelicidin (LL-37) [58] apparently in a negative feedback mechanism [59].

### 4.9. Mannose-Binding Lectin 2 (*MBL2*, 10q21.1)

Mannose binding lectin 2 (MBL2) is an important component of the first-line defense against infections. MBL2 acts as a pattern recognition molecule of a wide range of infectious agents recognizing sugar moieties such as mannose, N-acetyl glucosamine, fucose, and glucose present on the surface of several microorganisms, leading to their phagocytosis and MBL-associated protease-2-dependent activation of complement [60] through mannan binding lectin serine proteases (MASPs) such as MASP2 [61]. MBL also can induce phagocytosis in the absence of complement activation through an interaction with one or more collectin receptors [62] and is able to induce inflammatory responses by binding to receptors on phagocytes [60]. MBL deficiency, which mainly results from three relatively common single-point mutations in exon 1, reduces phagocytosis and internalization of intracellular pathogens protecting the host against intracellular infections such as leprosy and interestingly predisposes both to infection by extracellular pathogens and to autoimmune disease [60]. A significant negative association of MBL deficiency (<100 ng/mL) was observed with LL patients when compared with controls and TT patients, suggesting a protective role for MBL deficiency against the development of the most severe MB form of leprosy [63] explained because MBL deficiency could be advantageous against intracellular pathogens that exploit complement deposition on their surface to enhance uptake into phagocytes such as the case of *M. leprae* [14, 60]. SNP *G161A* was associated with protection from LL and *C154T *as well as *G170A* show no association in a population from Nepal [64]. The *LYPA* haplotype of *MBL2* was associated with susceptibility to leprosy *per se* and to progression to the lepromatous and borderline forms of the disease in subjects from southern Brazil [25]. Recently, it has been shown that MBL serum levels in leprosy patients are influenced by age, those patients aged >40 years had decreased MBL levels compared with patients aged ≤40 years. No association was found among analyzed SNPs in MBL2 with leprosy susceptibility or with any of its clinical forms [65].

### 4.10. Mannose Receptor, C-Type 1 (*MRC1*, 10p12.33)

The first genome-wide linkage leprosy study revealed a high LOD score in locus 10p13 among Indian populations [66]. Mannose receptor 1 gene* (MRC1 or CD206)* in this locus, specifically 10p12.33, codifies for mannose receptor C-type lectin (MR) on macrophages membranes, which recognize *M. leprae* cell wall mannose residues, as well as ovalbumin and zymosan. Notably, cellular entry through MR has been correlated with *M. tuberculosis *virulence [67]. From 75 analyzed SNPs in *MRC1* (101.8 Kb), *G396S* (*rs1926736*) was the only relevant. *396S* allele of *MRC1* is the only nonsynonymous polymorphism of exon 7 and was associated with protection against MB leprosy in 490 simplex and 90 multiplex Vietnamese families (704 patients). Similarly, but in haplotype analysis, when *399A* and *407F* polymorphisms are present, *396G *allele was also the risk factor in a case-control study of Brazilian patients with MB leprosy. It has been suggested that MR-*M. leprae* interaction is modulated by an additional accessory host molecule of unknown identity [68].

### 4.11. Nucleotide Oligomerization Domain 2 (*NOD2*, 16q12)

Nucleotide oligomerization domain 2 (*NOD2*, also called caspase recruitment domain-contanining protein 15, *CARD15*) is part of intracellular PAMPs known as NOD-like receptors (NLRs). Triggered by muramyl dipeptide recognition,* NOD2* interacts with the adapter protein RIPK2 (but not with pyrin domain containing-3, NALP3, another member of NOD-like receptor family) to initiate NF-*κ*B pathway signaling through the recruitment of the inhibitor of NF-*κ*B kinase (IKK) complex to the central domain of RIPK2 [6, 69].* NOD2* interacts with the autophagy-related gene *16L1 (ATG16L1*) and seems to be relevant inducing the autophagosome complex assembly (ATG5-ATG12) which induces autophagy in DCs leading to bacterial handling and generation of MHC class II antigen-specific CD4^+^ T cell responses in DCs. The polymorphisms *rs9302752* and *rs7194886* were associated with leprosy in Chinese population [26] but not in Indian or Mali population [70].

### 4.12. Parkin 2 and Parkin 2 Coregulated Gene (*PARK2* and *PACRG*, 6q25.2-q27)

*PARK2* (parkin) encodes an E3 ubiquitin ligase, with a role in the ubiquitin-proteasome pathway of intracellular protein degradation. *PARK2* also regulates the processing of protein antigens within macrophages, thereby affecting antigen presentation [35] and it is involved in cellular antioxidant response and T cell anergy [71]. *PARK2* was named for its association with an early-onset form of Parkinson disease [35]. *PARK2* is not only expressed in dopaminergic neurons but also in monocyte-derived macrophages and Schwann cells, primary host cells of *M. leprae* [71]. *PACRG* is a Parkin-coregulated neighbor gene of unknown function [12, 72] but together with *PARK2* have been proposed to be involved in degradation of TLRs and NF-*κ*B signaling pathway proteins [71].

In families from Southern Vietnam was reported a leprosy susceptibility region in 6p25. Of seven microsatellite markers underlying the linkage peak, alleles of two markers (*D6S1035* and *D6S305*) showed strong evidence for association with leprosy [72]. In a study of 197 simplex Vietnamese families, from 81 SNPs analyzed, 19 SNPs clustered in the 5′*PARK2 *and *PACRG* regulatory region and were significantly associated with leprosy. Common allele *T* of the first intron of *PACRG*, *PARK2_e01 (−2599*), and rare allele *C *of *rs1040079* were independently associated with an increased risk of leprosy and solely this two Tag SNPs explain the strong association in this region [12]. The association was reproduced in a Brazilian population (587 cases, 388 controls) where possession of as few of two of the 17 risk alleles was highly predictive of leprosy. Again, *PARK2_e01 (−2599*) and *rs1040079* were among the three most significantly associated SNPs in this population [12].

### 4.13. Solute Carrier Family 11, Member 1 (*SLC11A1*, 2q35)

*SLC11A1 *is expressed in spleen, lung, liver, and most intensively, in peripheral blood leukocytes [14]. *SLC11A1*(formerly *NRAMP1*) is a divalent cation transporter, when a live bacteria is phagocytosed. SLC11A1 protein is recruited to the macrophage phagosome membrane and limits pathogens' iron availability by exporting it from the phagolysosome [73, 74]. It has been suggested that* SLC11A1 *may influence the expression of MHC class II molecules, regulation of expression of *TNFA*, and induction of nitric oxide synthase [35]. By a genome-wide scan in Vietnamese families, it was found linkage with Mitsuda response in *SLC11A1* (2q35) and 17q21-25 loci, two regions previously linked to mycobacterial infection, granulomatous, [75] as well as early onset [76, 77] and severe asthma [78]. These similarities suggest that some common exacerbated epithelial responses may be regulated by *SLC11A1* and also by a major unidentified gene(s) in 17q21-25 [75]. The 3′UTR TGTG deletion in* SLC11A1* was associated with MB subtype in a Mali population and heterozygotes were more frequent among MB than PB patients [79, 80]. It has been proposed that allele 2 of the *SLC11A1* promoter is an independent genetic factor that predisposes cells to enable pathogen survival, probably due to its low efficiency in iron transport, but establishment of the infection and disease development are probably conditioned by additional genetic factors [81].

### 4.14. Toll-Like Receptor 1 (*TLR1*, 4p14)

Toll-like receptors are cell-surface molecules that play an important role in the recognition of pathogens [35, 82]. TRL1 : TLR2 heterodimers recognize triacyl lipopeptides from Gram-negative bacteria and mycoplasma, two of the three lipid chains of the triacylated lipopeptide interact with TLR2 and the third chain binds the hydrophobic channel of TLR1 and lead to NF-*κ*B activation [82]. The association between the hypofunctional TLR1 phenotype and protection against leprosy suggests that *M. leprae* may utilize TLR1 as part of its pathogenic mechanism [6]. The *T1805G (rs5743618 *or* I602S) *variant presumably downregulates *TLR1* expression on the cell surface and also causes abnormal signaling [83, 84] and has been associated with a decreased incidence of leprosy and with protection against reversal reaction [83] probably diminishing antimycobacterial cathelicidin (LL-37) expression [4, 5]. Allele *1805G* has been associated with diminished production of IL-1*β*, IL-6, and TNF in peripheral blood mononuclear cells stimulated with *M. leprae* [84, 85]. *TLR1 248SS* is associated with protection against leprosy [86].

### 4.15. Toll-Like Receptor 2 (*TLR2*, 4q32)

TLR2 is involved in the recognition of a wide range of pathogen-associated molecular patterns (PAMPs) as lipoarabinomannan from mycobacteria, lipopeptides from bacteria, peptidoglycan and lipoteichoic acid from Gram-positive bacteria, zymosan from fungi, tGPI-mucin from *Trypanosoma cruzi *and the hemagglutinin protein from measles virus [82], and in combination with MARCO and CD14 recognizes mycobacterial trehalose 6, 6′-dimycolate (TDM/cord factor) [87]. Lipoproteins trigger host responses via TLR2. TLR2 : TLR6 heterodimers are activated by diacylated lipoproteins from Gram-positive bacteria and mycoplasma and TRL2 : TLR1 by triacylated lipoproteins. Both heterodimers form a m-shaped structure and induce mainly inflammatory cytokines [82]. Furthermore, this increased ability to generate diversity in PAMP recognition-forming dimers with TLR1 or TLR6, TLR2 also has the ability to act together with other coreceptors on the cell surface that assist PAMP recognition. These include dectin-1, a C type lectin that binds fungus *β*-glucan and induces internalization and CD36, which act together with TLR2-TLR6 heterodimer to mediate the sensing of some but not all TLR2 agonists [82]. TLR-dependent IL-12p40 production was detected from primary human monocytes and monocyte-derived DCs after stimulation with a 19- or 33-kDa *M. leprae* lipopeptide [88]. However, some TLR2 nonsynonymous polymorphisms in Japanese patients were not associated with leprosy [89] probably because their variants are in a dispensable region of the receptor. Nonsynonymous substitution R677W of TLR2 gene was found in 10 out of 45 LL patients but absent in TT or nonlepromatous controls [90]. A study in Indian population reveals that the supposed mutation is in a pseudogene located 23 Kb upstream of TLR2, who shares 93% homology with its exon 3 [91]. A synthetic lipopeptide consisting of N-terminal portion of *M. leprae* 19-kDa lipoprotein triggered and increased the number of apoptotic Schwann cell line ST88-14 by a TLR2-mediated mechanism causing nerve damage [92]. Also, homozygosity for the 280-bp microsatellite in *TLR2* gene strongly increased the risk of RR in Ethiopian patients [93].TLR2 and TLR1 expression was relatively much weaker in LL skin biopsies [88].

### 4.16. Toll-Like Receptor 4 (*TLR4*, 9q32-q33)

TLR4 is a cell-surface receptor which recognizes respiratory syncytial virus fusion proteins, *Streptococcus pneumoniae* pneumolysin, mouse mammary tumor virus envelope proteins, and plant-derived cytostatic drug paclitaxel, also in complex with MD2+ engages bacterial lipopolysaccharide (LPS). There are two main possible routes of TLR4 activation, the direct route that leads to an early NF-*κ*B response and inflammatory cytokines expression and the indirect route through endosome leading to a subpathway of late NF-*κ*B activation and inflammatory cytokines profiles and to an alternative subpathway where Interferon Response Factor 3 (IRF3) induces type I IFN expression [82]. TLR4 is required for the host defense against *M. tuberculosis *and probably mediate response to mycobacterial heat shock proteins [4]. Furthermore, stimulation of monocytes with *M. leprae* partially inhibited their subsequent response to LPS. SNPs *896 G/A* (*D299G*) and *1196 C/T (T399I*) were associated with protection against leprosy in Ethiopic patients [94].

### 4.17. Tumor Necrosis Factor (TNF, 6p21.3)

Tumor necrosis factor is a proinflammatory and immunostimulatory cytokine secreted predominantly by monocytes/macrophages that has effects on lipid metabolism, coagulation, insulin resistance, and endothelial function [14]. High levels of circulating TNF (>1000 pg/mL) have been demonstrated in the plasma of some individuals with ENL who also develop erythema multiforme, a skin disease characterized by papular or vesicular lesions and reddening or discoloration of the skin often in concentric zones about the lesions [27]. *In vitro* peripheral blood mononuclear cells (PBMCs) from individuals with ENL secrete the highest amounts of TNF following stimulation with lipoarabinomannan, the main component of cell wall, when compared with other forms of the disease. It has been reported that half of BT patients have elevated serum levels of TNF [27].

### 4.18. Vitamin D Receptor (VDR, 12q12-q14)

Upregulation of the Vitamin D receptor gene (*VDR*) on macrophages is associated with increased intracellular killing of *M. tuberculosis. *In human monocytes and macrophages, the 25-hydroxyvitamin D3-1a-hydroxylase (CYP27B1) which converts the 25, D into the active 1, 25 form, upregulating and activating the vitamin D receptor and downstream induction of the antimicrobial peptide, cathelicidin [5] which is active against *M. tuberculosis *[95–98] and could be also active *in vivo* against *M. leprae,* but this deserves further investigation [2, 41]. In Indian population, homozygotes for the alternate alleles have been associated with LL and TT, respectively [99], and in Mexican population TT genotype of VDR obtained with *Taq*I (exon 9, codon 352) has been associated with LL [100].

## 5. Potential but Scarcely Explored Candidate Genes

### 5.1. Hydroxyacid Oxidase 1 Gene (*HAO1*, 20p12)

The marker *D20S115* in locus 20p12 that was associated with leprosy in Indian population [10] lies near atopic dermatitis with asthma loci (*ATOD3*) and also associated with psoriasis [101]. The closest gene to the marker *D20S115* in locus 20p12 (*∼*0.2 Mb) is *HAO1*, hydroxyacid oxidase 1 [102]. This enzyme is expressed predominantly in the liver and pancreas and utilizes a flavin cofactor to convert glycolate and 2-hydroxy fatty acids with the concomitant reduction of molecular oxygen to hydrogen peroxide [103]. It could be worthwhile to analyze its role in leprosy, especially in LL patients, whose lesions accumulate host-derived oxidized lipids [4], knowing that* M. leprae *has lost most of the genes involved in the detoxification of reactive oxygen and nitrogen species [104] and have a unique truncated hemoglobin [105, 106].

### 5.2. Toll-Like Receptor 6 (TLR6, 4p14)

TLR6 is a cell-surface receptor and TLR6 : TLR4 heterodimer with coreceptor CD36 recognizes oxidized low-density lipoprotein (ox-LDL), a known danger-associated molecular pattern (DAMP) [82]. TLR6 regulates *M. leprae*-Schwann cells interactions, and mainly in LL lesions, leads to phagocytosis and subsequent signaling for induction of lipid droplets (LDs or lipid bodies) biogenesis in infected cells, LDs favor mycobacterial survival and persistence, and thus are a hallmark of the foamy phenotype of *M. leprae*-infected Schwann cells [107]. LL lesions have a marked upregulation of host lipid metabolism genes when compared with TT lesions. Virchow cells (lepra cells or foam cells) are macrophages containing intracellular accumulation of a specific host-derived oxidized phospholipid, 1-palmitoyl-2-(5, 6-epoxyisoprostane E2)-sn-glycero-3-phosphorylcholine (PEIPC) [4]. Theoretically, if TLR6 is mutated at its heterodimerization site it could not form the TLR2 : TLR6, and the subsequent recognition of PAMPs or DAMPs should be abrogated. This epistasis should be considered in studies of TLR2 [82]. Biomedical relevant TLR6 polymorphisms remain to be discovered and a bioinformatic transcriptional impact could be used as a polymorphism sieving method [108]. 

### 5.3. Toll-Like Receptor 8 (TLR8, Xp22.3-p22.2)

TLR8 is expressed exclusively in intracellular vesicles such as the endoplasmic reticulum, endosomes, lysosomes, and endolysosomes, where it recognized microbial nucleic acids. TLR8 is expressed in various tissues with the highest expression in monocytes and is upregulated after bacterial infection. TLR8 mediates the recognition of R-848 and viral ssRNA but this function seems to overlap with TLR7 [82]. Four polymorphisms in TLR8 showed protection against TB [4] and it will be worthwhile to assess them in leprosy susceptibility. Furthermore, their localization in a sexual chromosome could be relevant in gender-associated susceptibilities (leprosy prevalence presents male: female ratio of 1.5–2.0 : 1) [19] and X-linked heritabilities, although no linkage of leprosy susceptibility with the X chromosome has been found to date [109].

## 6. Further Considerations in Leprosy Immunogenetics

### 6.1. Homo Sapiens-*M. Leprae* Genetic Interaction and Treatment Effect

With the above mentioned, it is now clear that most of the variants in clinical states in leprosy could be due to host genetics [2, 7, 8, 24, 110–112], and it is also relevant to study whether *M. leprae* variants [104, 110–116] and its genetic interaction with human genome accounts for some clinical variability, specially for drug-resistant leprosy in *M. leprae* genes *folP1, rpoB, *and* gyrA* which are associated with resistance to dapsone, rifampicin, and ofloxacin respectively [117, 118]. Also of relevant importance is the effect of immunosuppressive drugs on leprosy reactions [35].

### 6.2. Ethnic Susceptibility Differences due to Genetic Background and Technical Bias

It is relevant to mention that the ethnic genetic pool can affect susceptibility to disease [119] for example, the significant differences of polymorphism frequencies in *DEFB1* gene among Mexican population when compared with German and Chinese populations [46], which gene, as above mentioned was associated with LL [2]. It will be worthwhile to carry out susceptibility studies in other populations and be cautious with meta-analysis results in leprosy, because the relevancy of a gene in the disease could be influenced to allele/genotypes/haplotypes frequencies according to ethnic background [119, 120]. 

A relevant issue of concern is due to technical limitations, for example, the fact that commercial microarrays do not contain all relevant probes for SNPs associated with leprosy and many biomedical important polymorphisms are poorly represented. These technical limitations lead to a bias in the scientific interpretation of results and this issue is usually not mentioned in genome-wide association studies (GWASs) and authors still claim for a “complete genome” analysis [121]. 

## 7. Efficient Prevention, Fast Diagnostics, and Successful Therapeutics, the Goal of Translational Medicine: Leprosy as an Example

One potential application of genetic association studies' conclusions could be a starting point to a better understanding of disease by proposing candidate genes to be assessed with *in vitro* and *in vivo* studies, and also to evaluate the relative impact of genetic variants related to ethnicity versus environmental factors, in order to design better alternative diagnostics and therapeutics that could solve the public health problem that leprosy still represents.

Nevertheless, an imminent effect in patients with genetic testing information could be distress and anxiety so this information must be handled wisely by researchers and clinicians [122] because education plays a crucial role in the management of leprosy, is relevant to carefully explain to the affected individual and their family the infectious nature of the disease, and is curable [9]. Now with information availability from the ongoing 1000 Genomes [123, 124] and Human Variome projects the situation claims priority, and ethical standards must be established, reviewed, and updated constantly [125].

One recent and promising proposal in infectious diseases treatment comprising leprosy is the administration of antimicrobial peptide elicitors (APEs) specifically with the posibility of upregulating antimicobacterial host defense peptides as hBD-1 [41, 126] and cathelicidin LL-37 [127, 128] or the classical approach of APs direct administration [129], in the latter case with its inherent drawbacks [41]. Effectiveness and safety of APEs or APs in clinical practice is an exciting upcoming event. Even though the leprosy morbid map is far from complete, this is just one potential application that must be moved from bench to bedside.

## Figures and Tables

**Table 1:** Polymorphisms as predictors of leprosy clinical status and associated reactions. Most common human genetic polymorphisms irrespective of its ethnic origin [8, 21–31]. PB, paucibacillary; MB, multibacillary; RR, reversal reaction; ENL, erythema nodosum leprosum. Susceptibility alleles/genotypes/haplotypes are underlined, protection alleles/genotypes/haplotypes are not. (+), NS; associated but polymorphisms not specified; Ht, haplotype.

		WHO		Ridley-Joplin		Reactions		
Gene	*Per se*	PB	MB	TT	LL	Mitsuda	I (RR)	II (ENL)
17q21-25						(+), NS		
C13orf31/ CCDC122			*rs3764147*					
			*rs10507522*					
C3	<*1.1 mg/mL serum *							
C4A					*C4AF1*			
C4B								C4B*Q0
CFB								BfF
COL3A1			*250 bp/250 bp*					
CR1	4795 A/G							
CTLA4	104 bp							
DEFB1					668 *C*/G (−44, rs1800972)			
FCN2	Ht AGA (−986/−602/−4)							
HLA	A*0206, A*1102, B*4016, B*1801, B*5110, Cw*0407, Cw*0703, HLA-DRB1*1501		HLAB46/MICA-A5		Ht A*1102-B*4006-Cw*1502			
HSPA1				*HSPA1A-A*				
IFNG	Intron 1 CA repeat, +874T							
IL-4	−590 *T*/C							
IL-10	AGC, *TAC* (−3575/ −2849/−2763)	819 C/*T *						
	−819 TT	−592 C/*A *						
IL12B	3′UTR 1188 A/*C *							
IL-12RB2					−1035 A/*G *			
					−1023 A/*G *			
					−650 *del*/G			
					−464 A/*G *			
KIR				KIR2DS3				
LAMA2				7809 T/C				
LRRK2				*rs141938*				
LTA	−294, −293, +10 , +80, +252, +368							
LTA4H			rs1978331 T/C					
			rs2660898 T/G					
MBL2	*Ht LYPA (−550L/221Y/+4P/exon1A)*				*<100 ng/mL 161 G/*A			
MICA/MICB			MICA-A5					
MRC1	rs1926736 *G*/A (396S)		*Ht GAF (396/399/407)*					
			rs2437257 C/G (407L)					
NOD2	rs12448797 T/*C*, rs2287195 A/*G *						rs2287195 A/*G*, rs8044354 A/*G *	rs6500328 A/G
	rs8044354 A/*G*, rs13339578 G/*A *						rs8043770 C/*G*, rs7194886 C/*T *	rs7194886 C/*T *
	rs4785225 C/*G*; rs751271 A/*C * rs9302752/rs7194886						rs1861759 A/*C*, rs47852225 C/*G *	rs17312836 A/*C *
							rs751271 A/*C *	rs1861758 C/*T *
								rs1861759 A/C
PACRG								
PARK2	*−697 G, −2599 T, −3024 C, −3800 G*							
	rs1514343 T, rs1333955 C , rs1040079 C							
RIPK2			rs42490					
SLC11A1	allele 2		3′UTR TGTG del			(+), NS		
TAP		TAP2B						
TIRAP	180 S/L							
TLR1	T1805G, GG (I602S), 743 *GG *						T1805G, (I602S)	743 G
TLR2	microsatellite 290 bp						*280 bp/280 bp, 597 C/T*	
							*Ht 280/297C/1350 T*	
TLR4	896 *G*/A (D299G), 1530 G/T							
	1196 *C*/T (T399I)							
TNFA	(−308 G/A)	*−308 G/A*			*−308 G/A*			>1000 pg/mL
VDR					TT (TaqI)			
